# Tell or Not to Tell: Changes in Ukrainian Older Adults’ HIV Status Disclosure Practices During Intersecting Covid-19 and War Crises

**DOI:** 10.1007/s10461-025-04955-w

**Published:** 2026-02-24

**Authors:** Alexandra A. Deac, Katherine M. Rich, Irina Zaviryukha, Oleksandr Zeziulin, Tetiana Kiriazova, Valerie A. Earnshaw, Daniel J. Bromberg, Sheela V. Shenoi, Julia Rozanova

**Affiliations:** 1 Department of Health Services & Population Research, King’s College London, London, SE5 8AB UK; 2 Institute for Connected Communities, University of East London, London, E15 4LZ UK; 3 Harvard Medical School, Boston, MA 02115 USA; 4 European Institute of Public Health Policy, Kyiv, 04123 Ukraine; 5 Ukrainian Institute On Public Health Policy, Kyiv, 1054 Ukraine; 6 Department of Human Development and Family Sciences, University of Delaware, Newark, DE USA; 7 Section of Infectious Diseases, Yale University School of Medicine, New Haven, CT 06510 USA; 8 Centre for Interdisciplinary Research On AIDS (CIRA), Yale University School of Public Health, New Haven, CT 06510 USA

**Keywords:** HIV, Disclosure, Humanitarian crises, Older people

## Abstract

Humanitarian crises exacerbate challenges to accessing HIV care. Older people with HIV (OPWH, aged ≥ 50 years) may seek additional support with HIV care, which often requires disclosing their HIV status to healthcare providers, family, or friends. We sought to understand how crises, including COVID-19 and the war with the Russian Federation, have changed OPWH disclosure behaviours over time. We surveyed OPWH in Kyiv, Ukraine, by phone at four-time points: May–June 2020 (Wave 1), January–February 2021 (Wave 2), January–February 2022 (Wave 3) and May–June 2022 (Wave 4). Participants’ responses were compared longitudinally. The primary outcome was new HIV status disclosure, and the independent variables were living conditions (living alone, not living alone) and HIV care support. Other variables analysed were age, gender, comorbidities, social support, depressive and anxiety symptoms, time since diagnosis, and history of addiction. A mixed-effects multivariable logistic regression model was used to assess the relationship between HIV status disclosure and independent variables. Of the 123 participants recruited, 90 OPWH completed the survey across all four-time points, of which 46 (51.1%) were women, and the mean age was 54.3 (SD = 6.38) in Wave 1 and 58.8 (SD = 6.64) in Wave 4. Men were less likely to disclose their HIV status (adjusted odds ratio [aOR] = 0.29; 95% CI [0.09, 0.94], p = 0.04) as were OPWH living alone (aOR 0.29, 95% CI [0.10 – 0.85], p = 0.02). Other variables were not statistically significant (p > 0.05). Findings underscore the pressing need for interventions that prioritise comprehensive, inclusive, and tailored to address key barriers to HIV disclosure, such as gender norms and social isolation. Future HIV care and support programs should integrate targeted strategies to foster community support, mitigate stigma, and promote disclosure as a pathway to adherence, overall health outcomes, and well-being of OPWH, particularly those living alone or within marginalised subgroups.

**Supplementary Information:**

The online version contains supplementary material available at 10.1007/s10461-025-04955-w.

## Introduction

In Ukraine, older people living with HIV (OPWH), aged ≥ 50 years old, comprise a quarter of the people with HIV (PLWH) [1], and this number is expected to rise rapidly in the future [2, 3]. The overlap of the Covid-19 pandemic in December 2019 [4, 5] and the Russian invasion of Ukraine on 24 February 2022 [6] exacerbated challenges to accessing HIV care (e.g., as doctors were redeployed to fight against Covid-19 and clinic visits were reduced), which may have impacted the autonomy of OPWH in navigating and accessing their usual HIV treatment[7, 8]. There were increased reports of disruptions to antiretroviral treatment (ART) [9] [10]. Amid this complex context, which constitutes the contextual backdrop of this study referred to as ‘crises’, OPWH become potentially the most vulnerable yet invisible victims of disruption of health care services during humanitarian crises [11, 12].

Disclosure to family, friends or healthcare providers allows individuals to access necessary medical care, receive support for HIV and other diseases, and prevent transmission of HIV [13, 14]. Traditionally, OPWH have relied on close family and friends to support them with emotional and practical support, including transportation to medical centres and accessing HIV-related care [15]. However, the humanitarian crisis led to massive internal displacement and population outflow to neighbouring countries, estimated at nearly 25% of the population [16]. Mass migration left many OPWH living alone or cohabiting with strangers, complicating disclosure decisions. Many elderly individuals remained in their homes and were not evacuated, leading to a significant loss of close contacts and a breakdown in community ties [17]. Consequently, they became more dependent on formal healthcare services for social support due to the absence of family support, a situation worsened by deliberate attacks on healthcare infrastructure [17, 18]. Evidence suggests that OPWH are at an increased risk of social isolation and are less likely to disclose and seek help [19].

Disclosing one’s HIV status is a continuous decision-making process [19] requiring multiple decisions actively shaped by the perceived outcome of the disclosure, as well as socio-ecological and cultural elements [15, 20, 21]. According to the Disclosure Process Model (DPM) framework [22], the interconnectedness of antecedents to disclosure (e.g., gender, living conditions), disclosure events, and individual and distal outcomes (e.g., mental health, HIV care support) shape HIV disclosure patterns [23]. Living alone and lack of HIV care support have been associated with non-disclosure of HIV status [24]. Other barriers to HIV disclosure range from fear of negative reactions, stigma and discrimination to privacy concerns and social and cultural norms [19, 25, 26]. For example, a review of the available evidence suggests that older adults who receive an HIV diagnosis prefer to keep it undisclosed in order to achieve culturally appropriate goals for their stage in life. OPWH may strive, for example, to serve as role models or mentors to younger generations, transmitting wisdom and generativity, and fostering connections and support [27]. However, the fear of stigma or prejudices following disclosure could hinder their ability to do so [21], particularly in societies wherein acceptance and respect are tied to specific social norms [25, 28–31].

Facilitators of HIV disclosure among OPWH include regular and supportive interactions with healthcare providers, a sense of stability, protection and privacy, close relationships, living conditions, marital status and confidant’s HIV status [28–33]. For example, if their confidant (the person to whom they disclosed) is also HIV seropositive, OPWH may feel more inclined to disclose [22]. Evidence suggests that cohabitation and social support are associated with disclosure and adherence to treatment [29, 34]. Notably, studies have shown that there are gender disparities in the disclosure of HIV status [35, 36]. Women tend to face more severe stigma than men [35, 37], while men may avoid disclosing their status due to concerns about masculinity or being perceived as weak [38]. However, no study to date has sought to explore the impact of crises on HIV disclosure patterns among OPWH, a vulnerable group characterised by potentially increasing frailty, comorbidities, and food and financial insecurity [11].

This longitudinal study aims to bridge that gap by examining the impact of crises on HIV disclosure. Given crisis-related displacement and disruption of traditional multigenerational households, we sought to understand the impact of living conditions and HIV care support on disclosure behaviour and identify factors associated with HIV status disclosure. Guided by the DPM as our conceptual framework, we sought to examine changes in HIV disclosure behaviour among OPWH during the Covid-19 pandemic in Ukraine and the military conflict escalations from early 2020 to May 2022. This study adds to the existing knowledge on HIV disclosure during the Covid-19 pandemic and war (both compounding the humanitarian crisis in Ukraine).

## Materials and Methods

### Ethical Considerations

The study received ethical approval from the Institutional Review Board at Yale University (IRB Protocol #200,002,227) and the Ukrainian Institute on Public Health Policy in Ukraine (IRB Protocol #2017–021-08). Verbal informed consent was obtained from all participants before any data collection.

### Participant Recruitment and Data Collection

This study used four waves of data collected between May 2020- and June 2022. It was conducted with a cohort of OPWH receiving antiretroviral treatment (ART) at two treatment sites in Kyiv: the Kyiv City AIDS Centre and the ‘Sociotherapy’ Centre, which provides substance use treatment. Together, they serve approximately 15,000 PLWH annually. Participants were purposively recruited using convenience sampling and surveyed by trained staff via phone from May to June 2020 (Wave 1). Subsequently, the same participants were surveyed by phone by the same interviewer three more times: in January–February 2021 (Wave 2), January–February 2022 (Wave 3) and May–June 2022 (Wave 4). Eligibility criteria were: OPWH; currently registered for HIV care at one of the participating sites; being able to provide informed consent; and being willing to participate in a phone survey. The target sample was a minimum of 100 OPWH. To enable rapid situational analysis, clinical staff phoned as many potentially eligible participants as possible using internal records of patients in care. The recruitment process entailed the psychologist from the AIDS Center (recruiter) drawing a list of OPWH patients and their contact information from the medical database. Then, the recruiter contacted these OPWH by telephone to explain the study and offer participation. Since interviewers were HIV care workers, not only did they deeply understand OPWH concerns about confidentiality, but crucially for OPWH speaking with them, the interviewers did not disclose HIV status to an outsider, reassuring OPWH that their HIV status will not be disclosed to outsiders. This assurance was crucial in establishing trust, underscoring the interviewers’ credibility and their commitment to maintaining strict confidentiality regarding the HIV status of participants. After the patient agreed to participate, the recruiter agreed on a date and time to phone the participant and transferred their contact to the interviewer. The interviewer obtained verbal informed consent from the participant over the phone, and conducted the survey remotely.

The phone surveys were conducted by three female interviewers with clinical training and experience in HIV care in Ukraine, fluent in Ukrainian and Russian. The interviewers contacted the potential participants (via telephone) and secured verbal informed consent prior to administering the survey. There were no withdrawals following verbal consent. The interviewers were situated alone in private rooms to facilitate complete confidentiality. The interviewers used a device (computer or tablet) to navigate the survey items, and the questionnaire answers were recorded in the REDCap electronic research database*.* Each interviewer read the questions clearly according to the questionnaire structure with suggested answer options. All answers were recorded and saved by the interviewers in real time. Participants completed the 40-min survey (range: 30–60 min). They were compensated with 200 Ukrainian hryvnias (approx. $5 USD) as either phone credit or bank transfer (per participant’s choice).

**Fig. 1:**
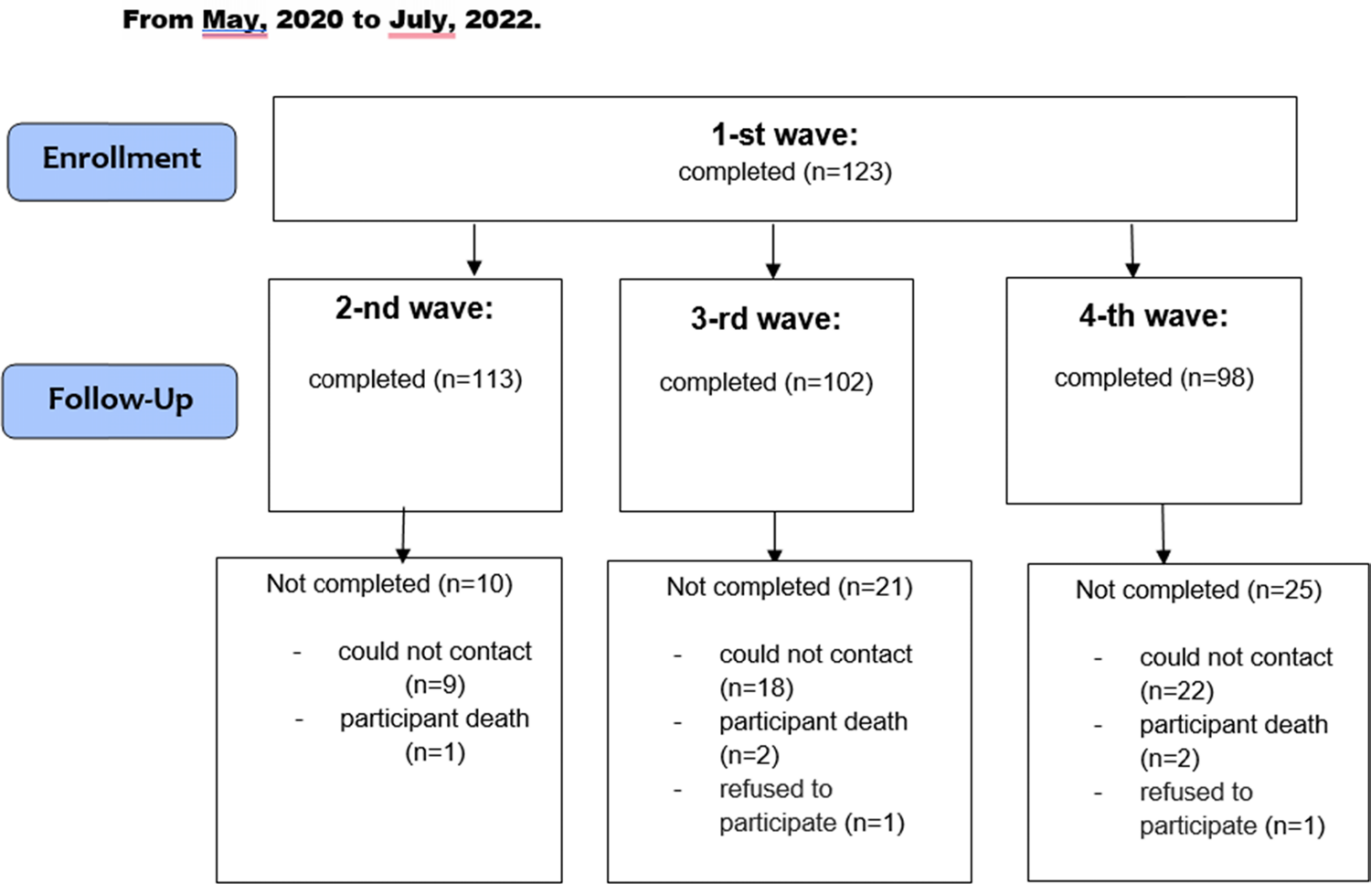
Participants flowchart

From 147 OPWH patients the recruiter phoned, 125 were successfully reached, and 123 agreed to participate. The first wave involved 123 participants, the second 113 (9 lost, one deceased), the third 102 (18 lost, one refused, 2 deceased), and the fourth 98 (21 lost, two refused, two deceased). Due to contextual low life expectancy among OPWH with comorbidities and to address recruitment challenges among this vulnerable population, during the 1 st wave, we included 19 ‘OPWH-candidate’ participants aged between 45 and 49 in the study. By the 2nd wave, this number decreased to 17, followed by 10 in the 3rd and 8 in the 4th waves. Efforts were made to reconnect with participants lost to follow-up using dedicated recruiters (Fig.1).

### Survey Development

The survey was developed incorporating insights from global mental health experts, including psychiatrists and clinical psychologists, who advised on including of various scales such as Patient Health Questionnaire-9 (PHQ-9), Generalized Anxiety Disorder-7 scale (GAD-7), and quality of life measures. After drafting, the survey was presented for feedback to an established community working group (CWG) in Ukraine comprised of OPWH and HIV and addiction providers, particularly on crafting non-scale questions and options for multiple-choice responses while keeping the standardised scales unchanged. Following the incorporation of this feedback, the revised survey underwent testing by members of the CWG, ensuring relevance, comprehensiveness, and usability for phone-based data collection. Based on literature review [39–44], and informed by the DPM framework and factors that influence the decision of HIV status disclosure (i.e., antecedents to disclosure) [23], variables were collected to capture five key domains: (1) socio-demographic characteristics (gender, living conditions), (2) HIV- related factors (HIV care support- defined as having someone who supports HIV treatment [i.e. provides reminders about medication] time since diagnosis), (3) history of addiction, (4) psychosocial and psychiatric conditions (social support, anxiety, depression), and (5) chronic conditions. The survey contained items previously validated in Ukraine or similar low- middle-income countries [39, 45–49], and we included both the PHQ-9 and GAD-7 scales per the Ministry of Health of Ukraine screening recommendations [50, 51]. The same instrument was used at all four-time points.

### Measures

#### General Characteristics

Participants were asked their gender, indicating whether they identify as male, female, transgender or “refuse to respond” option. Age was collected through self-report and validated using HIV Clinic Management Information System.

#### HIV Care

New HIV disclosure was defined as: “a new disclosure about HIV status to some person (who was important to the participant and who plays a significant and recurring role in the participant’s life, e.g., family, friends, healthcare provider) who was previously unaware of the seropositive status”. Participants were asked: “Have you told any new person about your HV status?” and follow up prompts were: “Whom did you tell about your HIV status? (partner/husband/wife; close friend; parents; child(ren); another family member; acquaintance; colleague; healthcare worker (besides your HIV doctor); other)”. HIV care support was operationalised as having someone (e.g., healthcare workers, family, friends) who supports HIV treatment (e.g., providing them with any support such as reminders about medication). Participants were asked:” Do you have somebody who over the last 6 months supported/helped you in treating HIV infection (provided reminders, controlled [taking medication)?”. The interviewer read out to participants examples of someone who reminds them to take medication or pick up their prescription. It was recorded as a binary response (yes/no). Time since HIV diagnosis was defined as the total number of years since participants received their HIV diagnosis for the first time. Participants were asked, “When did you find out you had HIV?”.

#### Drug and Alcohol Use

History of addiction was ascertained through self-report of a diagnosis of a substance use disorder or an alcohol use disorder. Notably, all individuals referred from ‘Sociotherapy’ struggled with drug and/or alcohol addiction, while the clinic also provides a full range of HIV services to patients; so, by definition, anyone recruited from ‘Sociotherapy’ would have a confirmed addiction diagnosis.

#### Psychosocial

Living conditions were ascertained through self-report, and participants were asked whether they lived alone or with family or friends. Social support, defined as the number of individuals OPWH reported feeling they could trust and turn to for help, was assessed through a 10-item version scale [45]. For example, participants were asked, “How many close people do you have now (i.e. people whom you trust and can ask for help)?”. The social support scale ranged from 0 (no close people) to 999 close people (higher social support). Higher scores reflect higher social support.

#### Depression and Anxiety

Levels of depression were measured using the PHQ-9, which contains nine questions with possible answers scoring anywhere between 0 (lowest point) to 27 (highest point). A score of 5 or above as mild depression, and a score of 10 or above was classified as severe depression. Levels of anxiety were measured using the GAD-7 item with seven questions with a score range from 0 to 21 (severe anxiety). A score of 5 or above as mild anxiety and 10 or above as severe anxiety. Higher scores on the PHQ-9 and GAD-7 indicated greater severity [52–54].

#### Chronic Conditions

Comorbidities (e.g., diabetes, heart disease, cancer, arthritis) were assessed through self-reports. Participants were asked, “Do you have other chronic diseases besides HIV infection?”.

### Data Analysis

The primary outcome was HIV disclosure, and it was classified as a binary (yes/no) response. The independent variables were living conditions analysed as a binary response (living alone, not living alone) and HIV care support. Time since the disclosure was assessed in years, and mean and standard deviations were calculated. Depression and anxiety scales were analysed as binary outcomes (no symptoms or mild to severe symptoms), with a score of ≥ 5 being used to define symptoms of anxiety or depression. We described participant characteristics using means and standard deviations for continuous variables and frequencies and percentages for categorical variables. Gender was assessed as a binary outcome (Male/Female). Social support was analysed as a continuous variable. History of addiction and comorbidities were analysed as binary (yes/no) variables.

De-identified data were analysed in R version 4.0.0 (R Core Team, 2020; Vienna: Austria). Repeat analysis of variance (ANOVA) tests were conducted to assess changes in characteristics across time points. To analyse changes across survey time points, the dataset was restricted to participants who had completed all four waves. Next, a mixed-effect logistic regression model was employed to examine the relationship between disclosure and the predictor variables. The model incorporated each participant ID and the survey time point (i.e., Wave 1, Wave 2, Wave 3, Wave 4) as random effect terms to account for potential correlations within individual participant records and to account for correlations within survey waves. Odds ratios (ORs), 95% confidence intervals (95% CI), and p-values were calculated. P-values less than 0.05 were considered statistically significant. All variables that were significant in bivariate testing were entered into a mixed-effects multivariable logistic regression model, and adjusted odds ratios (aOR), 95% CI and p-values were calculated. Due to its exploratory nature, the multivariable logistic regression was not adjusted for type 1 error [55, 56].

## Results

In Wave 1 (baseline), there were 62 (50%) women, and the average age was 55.3, with a standard deviation (SD) of 6.8. Thirty-six (29%) OPWH reported living alone, and 47 (38%) reported having someone who provided HIV support. At baseline, nearly 7 out of 10 participants (70%) reported having chronic conditions. Over a third of the cohort (n = 43, 35%) had symptoms of anxiety, and nearly half (n = 56, 46%) had symptoms of depression during Wave 1. Nearly half of the participants (51.5%) reported a history of addiction by Wave 4. Across four waves, disclosure rates among participants steadily increased: 72% in Wave 1, 88% in Wave 2, 93% in Wave 3, and 94% in Wave 4. The average time since diagnosis was 8.67 years (SD = 7.1) in Wave 1, naturally increasing over time (Table 1).

**Table 1:** Description of Participants, Waves 1-4

** Variable**	**Wave 1 (May-June 2020) ** **(n=123)**	**Wave 2 (January-February 2021)** **(n=113)**	**Wave 3 (January-February 2022)** **(n=102)**	**Wave 4 ** **(May-June 2022)** **(n=98)**
Age, years	55.3 (6.8)	55.3 (6.4)	56.4 (6.6)	56.9 (6.4)
Gender (Woman)	62 (50%)	56 (50%)	48 (47%)	47 (48%)
Living Alone	36 (29%)	30 (27%)	26 (25%)	14 (14%)
HIV Support	47 (38%)	36 (32%)	46 (45%)	25 (26%)
Chronic Condition	86 (70%)	71 (63%)	76 (75%)	78 (80%)
Anxiety Symptoms*	43 (35%)	25 (22%)	34 (33%)	58 (59%)
Depressive Symptoms**	56 (46%)	45 (40%)	40 (39%)	71 (72%)
Social Support	4.9 (5.1)	4.7 (3.6)	4.4 (3.5)	4.4 (2.9)
Disclosure	88 (72%)	98 (88%)	95 (93%)	92 (94%)
Time since diagnosis (years)	8.67 (7.1)	8.94 (7.3)	9.52 (7.3)	9.28 (7.4)
History of addiction	52 (42.3%)	52 (46.0%)	52 (51.0%)	51 (51.5%)

*GAD-7: Anxiety symptoms was defined as score ≥5;

**PHQ-9: Depressive symptoms were defined as PHQ score**≥**5

When restricting the analysis to individuals who completed all four survey waves (n = 90), HIV disclosure status differed significantly over time (p = 0.002), increasing from 75.6% at Wave 1 to 95.6% at Wave 4, with a considerable jump between Wave 1 when 68 (75.6%) OPWH reported new HIV disclosure versus 82 (91.1%) in Wave 2 (i.e., during the Covid-19 pandemic). No other variables studied differed significantly by timepoint (all p-values > 0.5). See Supplementary Table 2.

In bivariate modelling (please see Table 2 below), having a history of addiction (OR 3.34 [95% CI 1.02–11.20], p = 0.04), and years since HIV diagnosis (OR 1.11 [1.02–1.22], p = 0.01) were associated with an increased likelihood of HIV status disclosure. Men were less likely to disclose their HIV diagnosis than women (OR = 0.28 [0.09–0.88], p = 0.03). Individuals living alone were less likely to have disclosed their HIV diagnosis than those who were not living alone (OR = 0.22 [0.08–0.65], p = 0.01). Other variables were not statistically significant (p > 0.05). In multivariable modelling, men were found to be less likely to disclose their HIV diagnosis than women (aOR = 0.29 [0.09–0.94]), as were individuals who reported living alone (aOR = 0.29 [0.10–0.85]).

**Table 2:** Factors associated with disclosure of HIV among OPWH

Variable	Bivariate OR** **(95% CI)**	**p-value**	Multivariable aOR*** (95% CI)	**p-value**
Age	0.97 (0.88 -1.04)	0.30		
Man (woman = ref)	0.28 (0.09 – 0.88)	0.03	0.29 (0.09 – 0.94)	0.04
Living Alone	0.22 (0.08 – 0.65)	0.01	0.29 (0.10 – 0.85)	0.02
HIV Support	1.08 (0.45 – 2.62)	0.85		
Chronic Condition	1.67 (0.71 – 3.95)	0.24		
Anxiety Symptoms*	1.98 (0.79 - 4.99)	0.15		
Depressive Symptoms*	1.15 (0.49 - 2.75)	0.75		
Social support (continuous)	0.95 (0.86 – 1.06)	0.36		
History of addiction	3.34 (1.02 – 11.20)	0.04	2.16 (0.67 – 7.00)	0.20
Time since HIV diagnosis	1.11 (1.02 -1.22)	0.02	1.07 (0.98 – 1.17)	0.11

*Binary variable (GAD-7: Anxiety symptoms was defined as score **≥**5; PHQ-9: Depressive symptoms were defined as PHQ **≥**5**Odds Ratio ***Adjusted Odds Ratio

## Discussion

Among OPWH, HIV disclosure plays a crucial role in shaping health-seeking behaviours and treatment adherence [57]. However, research on HIV disclosure patterns among OPWH during societal crises has been missing. Seizing the unique opportunity provided by Ukraine, the present study is the first to explore HIV disclosure in the context of major societal disruptions and to examine the impact of living conditions and HIV care support on disclosure behaviour. We found that across time, OPWH in our sample increased both rates of cohabitation and overall disclosure of their HIV status, yet the biggest jump in disclosure was not after the Russian invasion but during the Covid-19 lockdowns.

Consistent with previous research highlighting gender differences in disclosure patterns based on various factors such as fears of abandonment, relationships, or perceived benefits of disclosure [32, 35, 36], our study revealed that men were less likely to disclose their HIV status than women at any time across all Waves. A potential reason for this may be stereotypes like ‘HIV is a disease of gay men’[58, 59] among older people, whose initial views about HIV/AIDS were formed back in the 1980 s by media headlines. In Ukraine, high-risk groups such as people who inject drugs, prisoners, and sex workers have historically been seen as the primary drivers of the HIV epidemic [60, 61], but being labelled as gay (and thus ‘pervert’) could be even more horrifying. Given these entrenched perceptions, OPWH may practice stigma-related non-disclosure, particularly if individuals fear being wrongly categorized into highly stigmatized groups. This warrants further exploration, especially in relation to ageism and evolving societal attitudes toward HIV.

The DPM suggests that individuals weigh the potential risks and benefits of disclosure, with avoidance goals—such as fear of stigma, loss of support, or threats to masculinity—playing a key role in their decision-making process [19, 25, 26]. It is possible that men in our study may have experienced deeply rooted gender norms that equate male strength with being free from illness. In a social context where masculinity is often linked to resilience and invulnerability, HIV status disclosure may be perceived as a threat to one’s social standing and identity, contributing to reluctance to disclose their HIV status [62]. This is consistent with men’s overall lack of engagement in the HIV care cascade [63, 64]. Alternatively, some men may have chosen not to disclose their status to protect their loved ones from the stigma associated with HIV or to avoid reinforcing their own stigmatized identity.

Another finding was that, during the crises, OPWH living alone were significantly less likely to disclose their HIV status to friends, family or healthcare providers compared to those who lived with others. This finding is consistent with extant non-crisis research suggesting that OPWH who live alone are less likely to disclose their HIV status and adhere to treatment compared to individuals with HIV living with others [1, 65, 66]. A recent meta-analysis examining the impact of armed conflict on HIV treatment outcomes suggested that HIV care falls to the bottom of the priority list in emergency responses, highlighting the vulnerability of PLWH [67]. The DPM suggests that reduced opportunities to seek social support, such as living alone, may impact serostatus disclosure [22]. In the case of OPWH during crises, contextual complexities such as living alone point to the need for comprehensive emergency support. For example, enhancing peer support and integrating HIV disclosure decision aids into disaster preparedness toolkits may be strategies to address living situations (alone vs with others) and social isolation and facilitate safe HIV disclosure.

Our study revealed that cohabitation increased over time. Through the DPM lens, OPWH in our study may have gauged the positive potential outcomes of disclosing to their cohabitants which increased their approach goals. Consequently, OPWH might have felt more inclined to disclose, especially if the cohabitant was HIV-positive [22]. However, the dynamics of OPWH relationships with cohabitants are often nuanced. While cohabitants offer company, they may not necessarily assist with HIV treatment [37] or their support may be bounded by limited experiential knowledge about HIV [68].

Another finding was that OPWH appeared to have progressively disclosed their HIV status over time; however, the biggest increase in disclosures occurred between Waves 1 and 2, i.e., during the height of the Covid-19 lockdowns, when people – and particularly older adults—were the most isolated. We speculated that participants may have aimed to seek HIV care support, mainly as reports of having HIV care support decreased over time. This aligns with evidence suggesting that disease progression (or anticipation thereof – which could occur during Covid-19 especially when and how it affects OPWH was yet completely unknown) and a greater need for HIV care support are associated with increased selective disclosure [34, 66]. During the Covid-19 lockdowns, OPWH may have re-evaluated potential outcomes, deciding to disclose their HIV status and viewing disclosure as a route to accessing care and expanding social networks during isolation [34, 69]. It is also possible that OPWH in our sample may have inadvertently disclosed their HIV status due to the socio-ecological context. Specifically, the delivery of ART via post (particularly during Covid-19 lockdowns) or the need to travel to the clinic may have led OPWH to feel forced to disclose.

The low endorsement of HIV care support could be interpreted as independent and confident self-management or a lack of a supportive network aware of their HIV status. Both interpretations were plausible, and the survey did not explore further or provide clarification on these distinctions. Potentially, the adverse contexts created obstacles for OPWH in accessing HIV care, which led to greater disclosure. Also, it is conceivable that OPWH who lived alone received HIV care support from individuals who did not reside with them, either in-person or through virtual means. Conversely, some OPWH living with others may not have had HIV care support. It is important to note that HIV care support question in the survey did not imply a value judgment or necessity. Instead, it inquired whether individuals had someone to aid them in HIV treatment, such as medication reminders or support.

These findings underscore the need to implement strategies that involve OPWH as part of support systems. These strategies may alleviate the shortage of caregivers and formal health services support, tapping into their shared wisdom of navigating complex decisions about HIV status disclosure. In line with the DPM framework, gauging positive disclosure outcomes may reduce stigma among OPWH and mitigate avoidance goals. It may also foster supportive environments and account for individual and contextual factors influencing the decision-making in HIV disclosure.

This study has several limitations. Expressly, the small sample size may limit the generalizability of the findings. Similarly, the use of self-reported data may be subject to social desirability bias and recall bias. Finally, the recruitment strategy around the clinic areas in Kyiv may limit the generalizability of the findings. By including only OPWH already receiving ART, our sample likely consisted of people who are more engaged in their HIV care and potentially more open to disclosing their HIV status. Future research must explore under what circumstances HIV disclosure happens among OPWH during crises and also with what outcomes – whether supportive or stigmatizing reactions. Additional qualitative research is necessary to explore the perceptions and role of disclosure goals, where OPWH could reflect on how they made decisions about disclosure of their HIV status during the crises. Anecdotal stories from OPWH suggest that especially during crises disclosures may be inadvertent or unanticipated, and tailored decision aids may help OPWH prepare in advance for such situations to handle them appropriately. Another limitation is the concurrent collection of variables, limiting causal inferences and temporal analysis.

## Conclusions

This study sheds light on HIV disclosure patterns among OPWH during the Covid-19 pandemic and the Russian invasion of Ukraine in 2022. We found that disclosure and cohabitation rates increased over time, and living alone and male gender were significant markers of non-disclosure. Findings underscore the pressing need for interventions that prioritise comprehensive, inclusive, and tailored to address key barriers to HIV disclosure, such as gender norms and social isolation. Future HIV care and support programs should integrate targeted strategies to foster community support, mitigate stigma, and promote disclosure as a pathway to adherence, overall health outcomes, and well-being of OPWH, particularly those living alone or within marginalised subgroups.

## Supplementary Information

Below is the link to the electronic supplementary material.

Supplementary file1 (DOCX 23 KB)
