# Supplementary material for: Tell or Not to Tell: Changes in Ukrainian Older Adults’ HIV Status Disclosure Practices During Intersecting Covid-19 and War Crises
Source: AIDS Behav. 2026 Feb 24;30(6):1673–82. doi: 10.1007/s10461-025-04955-w (PMC13303438; doi:10.1007/s10461-025-04955-w)
Supplement: Supplementary file 1 — Supplementary file1 (DOCX 23 KB) [file 10461_2025_4955_MOESM1_ESM.docx]

**Supplementary Tables**

**Tell or Not to Tell: changes in Ukrainian older adults' HIV status disclosure practices during intersecting Covid-19 and war crises.**

Alexandra A. Deac^1†^, Katherine M. Rich^2†^, Irina Zaviryukha^3^, Oleksandr Zeziulin^4^, Tetiana Kiriazova^4^, Valerie A. Earnshaw^5^, Daniel J. Bromberg^6^, Sheela V. Shenoi^6,7^, Julia Rozanova^1,6,7*^

*Affiliations*

^1^ Department of Health Services & Population Research, King's College London, SE5 8AB, London, United Kingdom

^2^ Harvard Medical School, Boston, MA 02115, USA

^3^ European Institute of Public Health Policy, 04123 Kyiv, Ukraine

^4^ Ukrainian Institute on Public Health Policy, 01054 Kyiv, Ukraine

^5^ Department of Human Development and Family Sciences, University of Delaware, Newark, DE, USA

^6^ Section of Infectious Diseases, Yale University School of Medicine, New Haven, CT 06510, USA

^7^ Centre for Interdisciplinary Research on AIDS (CIRA), Yale University School of Public Health, New Haven, CT 06510, USA

† Authors contributed equally to this manuscript

*corresponding author: Julia.rozanova@yale.edu, Tel.: +1.203.824.81.30, ORCID: 0000-0002-9971-6767

| **Supplementary Table 1.** Analysis variable dictionary | | |
| --- | --- | --- |
| **General Characteristics** | | |
| **Gender** | | Categorical: Women, Men, Transgender |
| **Age** | | Continuous |
| **Living Conditions** | | Binary : Living alone or Living with family/ friends |
| **HIV Care** | | |
| **Disclosure** | | Binary: Patient has disclosed HIV status to someone |
| **HIV support** | | Binary: Patient reports that they have someone who supports HIV treatment (i.e. provides reminders about medication) |
| **Time since diagnosis** | | Continuous: Total years since diagnosis |
| **Drug and Alcohol Use** | | |
| **Drug Use** | Binary: Any reported illicit drug use during Covid-19 lockdown | |
| **Addiction** | Binary: Patient either self-reported a diagnosis of a substance use disorder or an alcohol use disorder OR the patient was recruited as a patient of an addiction treatment clinic | |
| **Alcohol Use (AUDIT-C)** | AUDIT-C: Lower risk drinking (Score <3); Higher risk drinking (Scores $\geq3)$  Validation: Bradley et al., 2007; Frank et al., 2008. | |
| **Psychosocial** | | |
| **Social Support** | Patients self-report on the number of individuals they felt like they could trust and turn to for help. | |
| **Chronic Conditions** | | |
| **Health** | Binary: Patient has a chronic condition in addition to HIV, including kidney disease, diabetes, heart disease. | |

| Supplementary Table 2. Summary Analysis restricted to individuals who completed all study waves | | | | | |
| --- | --- | --- | --- | --- | --- |
| Descriptive Statistics: Wave 1 – 4 | | | | |  |
| Variable | **Wave 1 (n=90)** | **Wave 2 (n=90)** | **Wave 3 (n=90)** | **Wave 4 (n=90)** | **p-value** |
| Age, years | 54.3 (6.38) | 54.9 (6.39) | 56.0 (6.65) | 56.8 (6.64) | -- |
| Gender (Woman) | 46 (51.1%) | 46 (51.1%) | 46 (51.1%) | 46 (51.1%) | -- |
| Living Alone | 25 (27.8%) | 20 (22.2%) | 21 (23.3%) | 13 (14.4%) | 0.221 |
| HIV Support | 36 (40.0%) | 23 (25.6%) | 39 (43.3%) | 25 (27.8%) | 0.766 |
| Chronic Condition | 61 (67.8%) | 59 (65.6%) | 69 (76.7%) | 73 (81.1%) | 0.297 |
| Anxiety Symptoms* | 36 (40.0%) | 24 (26.7%) | 31 (34.4%) | 55 (61.1%) | 0.333 |
| Depressive Symptoms* | 46 (51.1%) | 41 (45.6%) | 39 (43.3%) | 66 (73.3%) | 0.771 |
| Social Support | 4.18 (3.03) | 4.24 (2.88) | 4.17 (3.05) | 4.13 (2.42) | 0.618 |
| Disclosure | 68 (75.6%) | 82 (91.1%) | 85 (94.4%) | 86 (95.6%) | 0.002 |
| Time since diagnosis (years) | 9.68 (7.32) | 10.4 (7.32) | 11.4 (7.32) | 11.7 (7.32) | -- |
| History of addiction | 51 (56.7%) | 51 (56.7%) | 51 (56.7%) | 51 (56.7%) | -- |
| * Binary variable (Mild - Severe);  *GAD-7: Anxiety symptoms was defined as score ≥5;  **PHQ-9: Depressive sx were defined as PHQ score≥5 | | | | |  |

| Supplementary Table 3. Bivariate modelling of disclosure | | |
| --- | --- | --- |
| Variable | **OR** | **p-value** |
| Age | 0.97 | NS |
| Man (woman = ref) | **0.28** | **0.03** |
| Living Alone | 1.56 | NS |
| HIV Support | 1.08 | NS |
| Chronic Condition | 1.67 | NS |
| Anxiety Symptoms* | 1.98 | NS |
| Depressive Symptoms*s | 1.15 | NS |
| Social support (continuous) | 0.95 | NS |
| History of addiction | **3.34** | **0.04** |
| Time since HIV dx | **1.10** | **0.01** |
| * Binary variable (Mild - Severe);  *GAD-7: Anxiety symptoms was defined as score ≥5;  **PHQ-9: Depressive sx were defined as PHQ score≥5 | | |

| **Supplementary Table 4.** Individuals who had disclosed HIV by Wave and Gender | | | | | | | |
| --- | --- | --- | --- | --- | --- | --- | --- |
| **Women** | | | | **Men** | | | |
| **Wave 1 (N=61)** | **Wave 2 (N=57)** | **Wave 3 (N=54)** | **Wave 4 (N=51)** | **Wave 1 (N=61)** | **Wave 2 (N=57)** | **Wave 3 (N=54)** | **Wave 4 (N=51)** |
| 48 (78.7%) | 50 (87.7%) | 54 (100%) | 49 (96.1%) | 40 (64.5%) | 48 (85.7%) | 41 (85.4%) | 43 (89.6%) |
